# Supplementary material for: Assessment of generative de novo peptide design methods for G protein-coupled receptors
Source: PLoS One. 2026 Aug 27;21(8):e0355549. doi: 10.1371/journal.pone.0355549 (PMC13521358; doi:10.1371/journal.pone.0355549)
Supplement: Supplementary file 1 [file pone.0355549.s001.pdf]

S1 Table. List of all 113 peptide-receptor complexes used to assess peptide prediction accuracy.

Receptor	Peptide	Receptor Class	PDB ID	cyclic	DOI
ACKR1	C-C motif chemokine 7	A	8jps	no	10.1016/J.CELL.2024.07.005
ACKR3	CXCL12α	A	7sk3	no	10.1126/SCIADV.ABN8063
ACKR3	Stromal cell-derived factor 1	A	7sk4	no	10.1126/SCIADV.ABN8063
ACTHR	ACTH	A	8gy7	no	10.1038/S41422-022-00751-6
ADA1A	ρ-Da1a	A	8hn1	no	To be published
AGTR1	AT118-H Nanobody	A	8th3	no	10.1038/S41589-024-01620-6
AGTR1	AT118-L Nanobody	A	8th4	no	10.1038/S41589-024-01620-6
AGTR1	angiotensin II	A	6os0	no	10.1126/SCIENCE.AAY9813
AGTR2	angiotensin II	A	6jod	no	10.1016/J.STR.2019.12.003
APJ	MM07	A	8xzh	yes	10.1016/J.CELL.2024.02.004
APJ	Single domain antibody JN241	A	6knm	no	10.1126/SCIADV.AAX7379
BKRB1	[des-Arg10]kallidin	A	7eib	no	10.1038/S41594-021-00645-Y
BKRB2	bradykinin	A	7f6h	no	10.1038/S41467-022-28399-1
BKRB2	kallidin	A	7f6i	no	10.1038/S41467-022-28399-1
C3AR	ALA-SER-LYS-LEU-GLY-LEU-ALA-ARG	A	8ia8	no	To be published
C3AR	EP141 peptide agonist	A	8j6d	no	10.1016/J.CELL.2023.09.020
C5AR1	C5a anaphylatoxin	A	7y64	no	10.1038/S41422-023-00779-2
CALCR	Calcitonin gene-related peptide 1	B1	9auc	no	10.1021/ACS.BIOCHEM.4C00114
CALCR	Calcitonin human	B1	7tyo	no	10.1126/SCIENCE.ABM9609
CALCR	San385	B1	8f0k	no	10.1038/S41589-023-01393-4
CALCR	amylin peptide	B1	7tyf	no	10.1126/SCIENCE.ABM9609
CALRL	Adrenomedullin	B1	6uus	no	10.1021/ACSPTSCI.9B00080
CALRL	adrenomedullin 2/intermedin	B1	6uva	no	10.1021/ACSPTSCI.9B00080
CCR1	CCL15(26-92)	A	7vl9	no	10.1038/S41589-021-00918-Z
CCR1	CCL15(27-92)	A	7vla	no	10.1038/S41589-021-00918-Z
CCR2	C-C motif chemokine 2	A	7xa3	no	10.1038/S41421-022-00403-4
CCR6	CCL20	A	6wwz	no	10.1038/S41467-020-16820-6
CCR8	CCL1	A	8u1u	no	10.1038/s41467-023-43601-8
CML1	chemerin C-terminal peptide	A	7ykd	no	10.1073/PNAS.2214324120
CML2	Retinoic acid receptor responder protein 2	A	8xgm	no	10.1371/journal.pbio.3002838
CML2	chemerin C-terminal peptide	A	8jjp	no	10.1371/journal.pbio.3002838
CRFR1	Corticoliberin	B1	6p9x	no	10.1016/J.MOLCEL.2020.01.012

CRFR1	Urocortin	B1	6pb0	no	10.1016/J.MOLCEL.2020.01.013
CRFR2	Urocortin	B1	7ts0	no	10.1038/S41467-022-33851-3
CXCR1	Interleukin-8	A	8ic0	no	10.1038/S41467-023-39799-2
CXCR2	Interleukin-8	A	6lfo	no	10.1038/S41586-020-2492-5
CXCR3	CXCL11	A	8hnk	no	10.1038/S41594-023-01175-5
CXCR4	CXC12	A	8u4o	no	10.1038/S41594-024-01397-1
CXCR4	Stromal cell-derived factor 1	A	8k3z	no	10.1016/j.celrep.2024.114578
CXCR4	vMIP-II	A	4rws	no	10.1126/SCIENCE.1261064
EDNRA	ENDOTHELIN	A	8hcq	yes	10.1038/s41467-023-36998-9
EDNRB	BQ3020	A	8xve	no	10.1038/S41421-024-00705-9
EDNRB	ENDOTHELIN	A	8iy5	yes	10.7554/ELIFE.85821
EDNRB	endothelin-3	A	6igk	yes	10.1038/S41467-018-07094-0
EDNRB	sarafotoxin S6b	A	6lry	yes	10.1016/J.BBRC.2019.12.091
GALR1	galanin	A	7wq3	no	10.1038/S41467-022-29072-3
GALR2	Galanin, Human	A	7xbd	no	10.1371/JOURNAL.PBIO.3001714
GIPR	gastric inhibitory polypeptide (human) - Incretin	B1	7ra3	no	10.1073/PNAS.2116506119
GLP1R	CHEMBL410972	B1	6x18	no	10.1016/J.MOLCEL.2020.09.020
GLP1R	Dual-agonist peptide P15	B1	8jis	no	10.1073/PNAS.2303696120
GLP1R	Exendin-P5	B1	6b3j	no	10.1038/NATURE25773
GLP1R	Pro-glucagon	B1	7lly	no	10.1038/S41467-021-27760-0
GLP1R	SAR425899	B1	8jir	no	10.1073/PNAS.2303696120
GLP2R	glucagon	B1	7d68	no	10.1038/S41422-020-00442-0
GLR	Glucagon derivative ZP3780	B1	8fu6	no	10.1016/J.CELL.2023.02.028
GLR	MEDI0382	B1	8jit	no	10.1073/PNAS.2303696120
GLR	NNC1702	B1	5yqz	no	10.1038/NATURE25153
GPR15	GPR15LG	A	8zqe	no	10.1038/S41421-024-00698-5
GRPR	Gastrin-releasing peptide	A	7w3z	no	10.1073/pnas.2216230120
KISSR	Kisspeptin-10	A	8xgs	no	10.1126/SCIADV.ADN7771
MC3R	γ-MSH	A	8ioc	no	10.1107/S0907444909042073
MC4R	α-MSH	A	7f53	no	10.1038/s41422-021-00552-3
MC5R	INTERMEDINE	A	8inr	no	10.1107/S0907444909042073
MCHR1	Melanin-concentrating hormone	A	8wwk	yes	10.1038/S41421-024-00754-0
MCHR1	pro-MCH	A	8wss	yes	10.1038/S41421-024-00679-8
MCHR2	pro-MCH	A	8wst	no	10.1038/S41421-024-00679-8
MRGX1	Conorfamide-Tx2	A	8jgb	no	10.1038/S41467-023-40705-Z
MRGX1	Proenkephalin-A	A	8dwc	no	10.1038/S41589-022-01173-6

MRGX1	bovine adrenal medulla peptide 8-22	A	8jgf	no	10.1038/S41467-023-40705-Z
MRGX2	Cortistatin 14	A	7s8l	no	10.1038/S41586-021-04126-6
MRGX2	substance P	A	7vdm	no	10.1038/S41586-021-04077-Y
MSHR	INTERMEDINE	A	7f4d	no	10.1038/S41422-021-00557-Y
MTLR	motilin	A	8ibv	no	10.1126/SCIADV.ADE9020
NK1R	substance P	A	8u26	no	10.1038/S41592-023-02082-9
NK2R	HIS-LYS-THR-ASP-SER-PHE-VAL-GLY-LEU-MET-NH2	A	7xwo	no	10.1038/s41421-022-00437-8
NK3R	Protachykinin-1	A	8jbh	no	10.1038/s41421-023-00564-w
NK3R	neurokinin B	A	8jbg	no	10.1038/s41421-023-00564-w
NK3R	senktide	A	8jbf	no	10.1038/S41421-023-00564-W
NMBR	NMB30	A	8h0p	no	10.1038/S41422-022-00743-6
NMUR1	Neuromedin U-25 (human)	A	7w53	no	10.1038/S41467-022-29683-W
NMUR1	Neuromedin-S	A	7w56	no	10.1038/S41467-022-29683-W
NMUR2	Neuromedin U-25 (human)	A	7w55	no	10.1038/S41467-022-29683-W
NMUR2	Neuromedin-S	A	7w57	no	10.1038/S41467-022-29683-W
NTR1	CHEMBL48981	A	6pwc	no	10.1038/S41422-019-0256-2
NTR1	JMV431	A	6yvr	no	10.1126/sciadv.abe5504
NTR1	JMV449	A	6os9	no	10.1038/S41586-019-1337-6
NTR1	neurotensin	A	8fmz	no	10.1021/ACS.BIOCHEM.3C00029
OPRK	dynorphin A-(1-8)	A	8f7w	no	10.1016/J.CELL.2022.12.026
OPRM	Nanobody-E	A	8qot	no	10.1101/2023.12.06.570395
OPRM	endomorphin-1	A	8f7r	no	10.1016/J.CELL.2022.12.026
OPRM	β-endorphin	A	8f7q	no	10.1016/J.CELL.2022.12.026
OPRX	nociceptin/orphanin FQ	A	8f7x	no	10.1016/J.CELL.2022.12.026
OX2R	Orexin-A	A	7l1u	no	10.1038/S41467-021-21087-6
OXYR	oxytocin	A	7ryc	yes	10.1038/S41594-022-00728-4
PACR	[Ac-His1]PACAP-27	B1	8e3x	no	10.1038/S41467-022-34629-3
PACR	maxadilan	B1	6m1h	no	10.1038/S41422-020-0280-2
PAR1	Tethered agonist	A	8xor	no	10.1038/s41422-024-00997-2
PRLHR	Prolactin-releasing peptide PrRP20	A	8zps	no	10.1038/S41421-024-00724-6
PTH1R	Long-acting parathyroid hormone analog	B1	6nbf	no	10.1126/SCIENCE.AAV7942
PTH1R	PTHrP-(1-36)	B1	8haf	no	10.1038/S41401-022-01032-Z
PTH1R	Parathyroid hormone	B1	8flq	no	10.1016/J.STR.2023.04.002
PTH2R	TIP39	B1	7f16	no	10.1073/PNAS.2101279118
QRFPR	Orexigenic neuropeptide QRFP	A	8wz2	no	10.1038/S41421-024-00670-3

RL3R2	Insulin-like peptide INSL5 B chain	A	7yj4	no	10.1038/S41467-023-36182-Z
SCTR	SECRETIN	B1	6wzg	no	10.1038/S41467-020-17791-4
SSR2	SOMATOSTATIN	A	7t10	yes	10.1038/S41594-022-00727-5
SSR4	SOMATOSTATIN	A	7xms	yes	10.1038/S41422-022-00679-X
SSR5	Cortistatin	A	8x8l	yes	10.1073/pnas.2321710121
V2R	vasopressin	A	7dw9	yes	10.1038/S41422-021-00480-2
VIPR1	[Ac-His1]PACAP-27	B1	8e3y	no	10.1038/S41467-022-34629-3
VIPR2	PACAP-27	B1	7vqx	no	10.1038/S41467-022-30041-Z
XCR1	Lymphotactin	A	9ast	no	10.1073/PNAS.2405732121
