# Supplementary material for: Assessment of generative de novo peptide design methods for G protein-coupled receptors
Source: PLoS One. 2026 Aug 27;21(8):e0355549. doi: 10.1371/journal.pone.0355549 (PMC13521358; doi:10.1371/journal.pone.0355549)
Supplement: Supplementary file 2 [file pone.0355549.s002.pdf]

S2 Table. List of the hotspot residues selected for peptide generation.

PDB ID	Hotspot residues	GPCRdb numbering
6jod	T125	3.33x33
	M128	3.36x36
	K215	5.42x43
	F272	6.51x51
6lry	K182	3.33x33
	L257	45.52x52
	K273	5.38x39
	R343	6.55x55
	K346	6.58x58
8f7x	Y131	3.33x33
	V279	6.51x51
	V283	6.55x55
	Y309	7.43x42
