# Supplementary material for: Assessment of generative de novo peptide design methods for G protein-coupled receptors
Source: PLoS One. 2026 Aug 27;21(8):e0355549. doi: 10.1371/journal.pone.0355549 (PMC13521358; doi:10.1371/journal.pone.0355549)
Supplement: Supplementary file 3 [file pone.0355549.s014.pdf]

Please note that all line locations refer to the markup version of the manuscript.

Response to Reviewer 1:

1. Training data leakage must be addressed in the main text
"The authors acknowledge that endothelin/sarafotoxin complexes were present in the training sets and that performance degrades for post-cutoff structures (Supplementary Fig. S1, p < 0.001). However, the main results are not stratified by training-set membership or structural redundancy. The bimodal DockQ distribution is consistent with possible memorization and should be analyzed directly. Headline performance numbers, such as the reported Boltz-2 median DockQ and BoltzGen generation precision, should therefore be qualified until in-training and post-cutoff examples are separated. The authors should: (a) annotate main figures by training-set membership, (b) report separate summary statistics for in-training, homologous, and post-cutoff structures, and (c) qualify abstract-level performance claims accordingly. Both aggregate and stratified numbers are useful, but the prospective performance estimate should be clearly distinguished from potentially training-exposed performance. If the high-DockQ mode is enriched for in-training or closely homologous complexes, this should be explicitly stated."

Respose:
We want to thank the reviewer for this highly relevant comment. We agree that we did not elaborate sufficiently on the effect of training set inclusion and that our statements raised more questions than they did answer. In order to address this, we stratified our dataset by release date of the specific PDB IDs as well as homologous deposits of the identical receptor-peptide pair. We decided to keep the initial Fig. 2 (now Fig. 3) for visual clarity and summarized the DockQ scores for pre and post cutoff PDB IDs which were added to the Supplement as S3 Fig. We further edited the manuscript accordingly to address this.

Edit locations:
Page 12, lines 239-256;
Page 30, lines 534-538 in the revised, marked-up manuscript.
S3 Fig.

2. PAE aggregation method must be specified
"The paper identifies confidence miscalibration or overconfidence in incorrect peptide placements as a central problem, but does not specify whether PAE values reported are min, mean, or median over the inter-chain block. This is a critical methodological detail: min-PAE will be optimistically biased for peptides where even one residue is well-placed, while mean-PAE is sensitive to outlier misplacements. The aggregation method should be clearly stated in Methods, and ideally a supplementary comparison of aggregation strategies should be provided."

Respose:
Thank you for highlighting this methodological detail. We edited our manuscript to clarify that we averaged PAE values by taking the mean over all PAE values for the respective chain subset.

Location:

Page 19, lines 339-340 in the revised, marked-up manuscript.

3. Nanobodies should be separated from peptide statistics

"The dataset includes eight nanobody/sdAb complexes alongside short peptides and peptide hormones. Nanobodies have a stable β-sandwich scaffold that fundamentally changes the prediction task. This is not merely a size issue; it changes the physical problem from flexible peptide docking/design to prediction of a structured protein-domain interface. DockQ, fnat, and iRMSD behave differently for structured proteins vs. flexible peptides, and mixing them may distort aggregate statistics unless analyzed separately. Results should be stratified by size class (e.g., peptides ≤ 50 AA vs. protein ligands > 50 AA vs. nanobodies), and the abstract should state the dataset composition explicitly."

Respose:

We want to thank the reviewer for this objection. While we remain confident with our initial assumption that peptides, hormones and the loops of nAbs share comparable interaction characteristics with GPCRs, we revisited the data and confirm that the prediction tools indeed perform differently for larger ligands. We therefore modified Figure 5 and added Supplementary Fig. S2. We adapted the manuscript accordingly.

Location:
Page 7, lines 158-160;
Page 11, lines 223-227;
Page 13, lines 271-275 in the revised, marked-up manuscript.

4. The design workflow needs a diagram and parameter documentation

"For a benchmark paper, reproducibility is paramount. Critical parameters are currently scattered across Methods and Supplementary materials. The paper should include: (a) a main-text pipeline diagram showing the end-to-end workflow for each method, and (b) a consolidated table of all method-specific parameters with values used. Hotspot residue identifiers should be listed explicitly (not only shown in Supplementary Fig. S4). ProteinMPNN was apparently used at temperature 0.05, which is near-deterministic. The authors should justify this choice and discuss whether broader sequence sampling would alter the rescue analysis."

Respose:

We appreciate the comments regarding methodological clarity.
(a) We agree that a workflow diagram significantly improves clarity of the manuscript. We added a pipeline diagram to visualize the workflow of the two parts of our benchmark as Figure 1.

Location:
Page 6, lines 137-148 in the revised, marked-up manuscript.

(b) We agree with the reviewer that for the sake of reproducibility, method parameters need to be clearly stated. Instead of a table, which became too large to be presented visually appealing, we opted to revise the Methods section as we purposefully kept the majority of settings default. We further highlighted those parameters that we modified and refer to the methods documentation as well as our exemplary scripts in the accompanying zenodo.

For these changes see:
Page 32, lines 599-602 and the remainder of the Methods section in the revised, marked-up manuscript.

Additionally, we agree that the previous hotspot-related figure was insufficient. We added S2 Table containing the hotspot residue identifiers and their respective Ballesteros-Weinstein numbering in the SI as S2 Table

Further, the reviewer raises a very valid point as sequence composition is the final determining factor in designing novel peptides. The reasoning behind a temperature of 0.05 was that increasing diversity would require a larger number of sequences to be evaluated, which would shift the focus of the benchmark. We expanded our discussion with respect to ProteinMPNN in our manuscript.
For these changes please refer to Page 26, line 463 and Page 30, lines 552-558

5. ProteinMPNN "rescue" requires a circularity caveat
"The observation that ProteinMPNN sequence redesign improves DockQ scores is measured by re-predicting with the same structure prediction tools. This creates a circularity risk: MPNN produces sequences that structure predictors find easier to fold, not necessarily sequences that would physically bind. The improvement should be described as improved in silico recoverability or predicted placement, not as evidence of physical rescue or binding. The authors should explicitly state that independent physics-based validation would be needed to confirm binding."

Respose:
Thank you for pointing out this critical detail inherent in virtually all currently available ML-based pipelines. We agree that our initial wording needed improvement and we adapted the discussion respectively to include these arguments (see Page 29, lines 552-558)

6. GPCR terminology correction
"Line 93: GPCRs are described as "the largest group of proteins in the human genome." This is inaccurate or at least imprecise — GPCRs are generally described as the largest family of membrane receptors, not the largest group of proteins overall. Zinc-finger proteins, for example, are often considered a larger protein superfamily. Please correct."

Respose:
We thank the reviewer for highlighting this inaccuracy. This was indeed not the intended phrasing and we adapted the manuscript accordingly.

Location:
Page 4, line 97

7. Top-k success metrics
“The paper would benefit greatly from practical design-workflow metrics: best-of-N DockQ, top-1 and top-5 by confidence, and enrichment of acceptable designs among top-ranked candidates. The current aggregate distributions obscure the question practitioners care about: how many predictions are needed to find one good candidate, and can confidence identify it?”

Respose:
We appreciate this suggestion and confirm that reporting metrics of top-1 candidates holds great value and that allowed us to strengthen our benchmark. We adapted the corresponding Figures (now Fig. 6 and S5 Fig) and addressed it in the manuscript. We further discuss that a general number of predictions required to find a good candidate cannot be sufficiently provided within the scope of this paper as these numbers are highly target- and method-dependent and we would argue that 50 predictions already is a sufficiently large number.

Location:
Page 18, lines 325-333
Page 28, lines 497-499
Page 30, line 539

8. Receptor conformation awareness
“While the authors appropriately use holo templates from the same PDB entry, it would be helpful to note in the Discussion that real design campaigns will not have access to the cognate holo receptor structure. A brief discussion of how receptor conformational uncertainty (active vs. inactive state, G-protein vs. arrestin stabilization, engineered constructs) affects the transferability of these benchmark results would strengthen the paper’s practical relevance.”

Respose:
We want to thank the reviewer for pointing out this crucial fact regarding GPCR drug discovery. We connect the conformational uncertainty with the likelihood of memorization and discuss how these two aspects might prevent succesfull results in real drug design campaigns.

Location:
Page 30, lines 534-538

9. Membrane context for generation targets
“The paper notes that many RFdiffusion3 designs are placed outside the binding pocket in contact with membrane-spanning residues (84.5% for AT2). However, no formal membrane boundary is defined. For the three generation targets, adding OPM-based membrane planes and classifying failures as “membrane-incompatible” vs. “wrong pocket” would strengthen the analysis.”

Respose:
We want to thank the reviewer for this relevant remark. We revised our initial method to determine the placement of designed peptides, i.e. through the distance to hotspots and

RMSD to native and concluded that these were insufficient in precisely determining the location, i.e. “in pocket” or “in membrane”. We therefore performed a more thorough contact analysis and were able to update our previous percentages. We adapted the manuscript accordingly. The updated numbers and a depiction of the OPM membrane definitions are summarized in Supplementary Fig. S5.

Location:
Page 24, lines 419-421

10. Peptide chemistry stratification
“The dataset mixes linear, disulfide-constrained, and cyclic peptides. Constrained peptides have reduced conformational entropy and may be systematically easier to predict. A supplementary stratification by constraint class would be informative.”

Respose:
Thank you for this comment. We stratified by cyclic constraint and added S4 Fig and addressed it in the manuscript.

Location:
Page 11, lines 227-229
Added S4 Fig to the supplement

11. DockQ supplementation
“DockQ does not capture pharmacophore-level correctness. For the three generation targets, supplementary analysis of peptide N/C-terminal orientation and key hotspot contact recovery would connect the structural metrics to biological function.”

Respose:
We agree with the reviewer that the pharmacophore-level correctness is a highly relevant piece of information, especially with regard to actual drug discovery campaigns. However, during our initial analysis we realized that N/C-terminal orientation (such as flipped termini) is difficult to generalize sufficiently, as designs often did not end up with a linear N- or C-terminal entry deep into the pocket, especially for the cyclic representative Sarafotoxin S6b. We further believe that in true de novo peptide design campaigns novel peptide orientations are desired and their correctness cannot be solely described through terminal orientation.

12. Steric clashes and refinement
“Clash rates are computed on raw outputs without minimization (noted in Fig. 6 caption). Given that the authors recommend Rosetta refinement in the Discussion, relaxing a small representative subset (e.g., 100 structures per method) and reporting the change in clash rate would substantially strengthen the practical relevance of the benchmark.”

Respose:
We fully agree with the reviewer that the outcome of Rosetta refinement is of great interest. We opted to leave those calculations out of this benchmark for different reasons: a) we wanted to keep the benchmark in the scope of ML-based methods. b) the strength of

Rosetta refinement has been reported extensively before and adding it to this benchmark would not provide significant new insights. c) while we expect that Rosetta resolves both minor and severe clashes, the actual extent of this is highly dependent on the protocols used, i.e. minimization vs. relaxation, restricting vs. allowing movement of the target or the use of membrane/peptide specific scoring functions. In order to still provide Rosetta specific instructions we adapted the manuscript and refer to a selection of Rosetta-specific literature.

Location:
Page 31, lines 571-572.

Response to Reviewer 2:

1. "I think tha authors should include models and design in the zenodo, not only the PDBs in the dataset."

Respose:
We agree with the reviewer that in order to guarantee full transparency, output data has to be included in the zenodo. We adapted the repositories respectively and also appended it with exemplary scripts necessary for reproduction.

1. "Please specify the versions of all software packages and tools used in the study. In addition, clarify the programming language(s) used for custom scripts."

Respose:
We want to thank the reviewer for pointing out the lack of documentation regarding the software package version used in this work. We amended the method section to clarify this. Since the main methods used in this work depend on a large number of supplementary packages, we referred to the respective installation protocols.

Location:
Page 37, lines 701-721

2. "The manuscript mentions 124 complexes; however, it appears that some receptors are represented by multiple PDB entries. Please clarify how redundant or highly similar receptor structures were handled in the analysis. For example, structures such as 7SK3 and 7SK4 are very similar. Were both included independently? If so, how was potential bias or overlap in the comparison of generated designs addressed when the receptor is repeated?"

Respose:
We want to thank the reviewer for this correct observation. Based on this comment, we revisited our dataset composition and identified further occurrences of duplicate entries that went unnoticed previously due to inconsistent naming in the GPCRdb. Keeping these would scew statistics and we therefore removed duplicates and updated the downstream analyses and figures. This reduced the dataset from 124 dimers to 113 dimers. Accordingly, Figure 2 was adapted to include these changes.

Location:
Page 1, lines 33-34
Page 9, line 185 (Fig.2)

3. "It would be helpful to include an additional column in Supplementary Table S1 indicating the GPCR class of each receptor for easier interpretation of the dataset."

Respose:
We agree that this is helpful information and added the column to Supplementary Table S1.

Location:

Supporting Information, S1 Table.

4. “In figure 1, the manuscript states that were used 76 Class A ligands plus 31 Class B1 ligands, which sum to 107 rather than 124. Please clarify this discrepancy, as I may have misunderstood the dataset composition.”

Respose:
Thank you for addressing this issue. We revised the manuscript to emphasize the many-to-many relationship of GPCRs and their peptides, where one peptide can bind to multiple GPCRs and one GPCR can accept binding of multiple peptides, resulting in a larger sum of complexes than the sum of receptors alone.

Location:
Page 7, line 168-174.

5. “In Figure 2, I suggest including an additional plot for LRMSD to complement the current structural evaluation metrics.”

Respose:
We thank the reviewer for this suggestion. While we initially observed no significant difference between iRMSD and LRMSD, we reworked our analysis and realized that this remains a relevant piece of information. We therefore added Supplementary Fig. S2 and S3, which include every component of the final DockQ score.

Location:
Page 13, lines 271-275.
Supporting Information, S2 Fig. and S3 Fig.

6. “For the DockQ analysis shown in Figure 3, it would be useful to include representative structural examples illustrating extreme cases. For instance, showing one example with very low DockQ and another with broader interface agreement would help readers better understand how iRMSD and FNAT contribute to the final DockQ values.”

Respose:
Thank you for this remark, we fully agree that structural examples would significantly improve the reader's ability to interpret the DockQ score. We added such an example in S1 Fig.

7. “I believe the conclusions section would benefit from a more explicit discussion regarding scoring and evaluation metrics. At present, the take-home message about which scoring approaches are most informative or reliable remains somewhat unclear.”

Respose:
We agree with the reviewer that our initial phrasing might sound hesitant when it comes to selecting the most informative scoring approach. In fact, revising our analysis gave us a clearer picture and allowed us to rephrase our discussion more strongly.

Location:

Page 28, lines 495-499.

8. “In the “Comparing the Sampling of BindCraft, BoltzGen and RFdiffusion3” section, it would also be interesting to evaluate sequence or structural recovery metrics and discuss these results in the conclusions. Are the designs similar to the native peptides? This could provide additional insight into how well the generated peptides preserve relevant interaction features or binding determinants.”

Respose:
We want to thank the reviewer for this suggestion. Our benchmark has a strong focus on structural comparison and the impact of sequence composition was not covered in similar detail. We calculated sequence recovery for all sequences generated which allowed us to strengthen our arguments.

Location:
Page 24, lines 435-437
Page 26, lines 471-473
S8 Fig.
