# Supplementary material for: Assessment of generative de novo peptide design methods for G protein-coupled receptors
Source: PLoS One. 2026 Aug 27;21(8):e0355549. doi: 10.1371/journal.pone.0355549 (PMC13521358; doi:10.1371/journal.pone.0355549)
Supplement: Supplementary file 4 [file pone.0355549.s015.pdf]

Please note that all line locations refer to the markup version of the manuscript.

Response to Reviewer 1:

"*In the Discussion you recommend Rosetta refinement to resolve clashes. While I accept that you kept new calculations out of scope, the XML protocol file (or a fully reproducible RosettaScripts protocol) does not appear to be available in the Zenodo repository or supplementary materials. For a benchmark paper that already emphasizes reproducibility, it would be very helpful if you could either deposit the exact XML script you have in mind or provide a clear, ready-to-run protocol (ideally with the ref2015 energy function and appropriate membrane terms).*

It is also necessary to make sure the right citations are used so in the methods for rosetta the ref2015 should be properly cited. Citing only the general development papers is not enough for readers to actually reproduce or adapt the suggested step.

Please check that ALL scripts and files are provided for fellow researchers.

With that those addition I'm happy to recommend acceptance."

Response:
We note the reviewer's wish for a reproducible RosettaScripts XML protocol and the corresponding citation for the ref2015 energy function. An exemplary XML script, configured with the ref2015 energy function and applicable membrane terms, has been added to our zenodo. Furthermore, the manuscript has been revised to include the specific citation for the ref2015 energy function.

Response to Reviewer 2:

“*The total complex are 113. However, the sum of classes is not equal to 113 (58 class A +14 class B1). The author should explain this or make a correction in the text*”

Response:

Dear Reviewer 2, we would kindly refer to page 7, lines 164-174 in the manuscript where we highlighted the many-to-many relationship between receptors and their cognate peptides:

[...] The final dataset consists of 74 peptides binding to 58 class A receptors and 26 peptides binding to 14 class B1 receptors, reflecting the promiscuous many-to-many binding relationship that is characteristic for many GPCRs and their endogenous peptides (Fig. 2A). Prominent examples for this promiscuity in our dataset are the Glucagon-like peptide-1 receptor (GLP1R), amounting to five unique GLP1R-ligand complexes, or the Endothelin receptor type B (ET_B) with four unique ET_B-ligand complexes. Promiscuous ligands on the other side usually occur in the dataset fewer in numbers and mostly within a subfamily of receptors, e.g. Ang II binding to both Angiotensin II receptor type 1 (AT_1) and type 2 (AT_2) or Substance P binding to both Tachykinin receptor 1 (NK_1) and Mas-related G protein coupled receptor member X2 (MRGPRX2). [...]
