# Supplementary material for: Assessment of generative de novo peptide design methods for G protein-coupled receptors
Source: PLoS One. 2026 Aug 27;21(8):e0355549. doi: 10.1371/journal.pone.0355549 (PMC13521358; doi:10.1371/journal.pone.0355549)
Supplement: Peer review history

# Author comment: Author response to Decision Letter 0

**DOI:** 10.1371/journal.pone.0355549.r001

**Transfer Alert**

This paper was transferred from another journal. As a result, its full editorial history (including decision letters, peer reviews and author responses) may not be present.

17 Mar 2026

# Peer review: Decision Letter 0

**DOI:** 10.1371/journal.pone.0355549.r002

**Authors:** Shengwei Sun

13 May 2026

*De Novo*

Dear Dr. Junker,

Thank you for submitting your manuscript to PLOS ONE. After careful consideration of the reviewers' comments, we feel that it has merit but does not fully meet PLOS ONE’s publication criteria as it currently stands. Therefore, we invite you to submit a revised version of the manuscript that addresses the points raised during the review process.

Please submit your revised manuscript by Jun 27 2026 11:59PM. If you will need more time than this to complete your revisions, please reply to this message or contact the journal office at plosone@plos.org. When you're ready to submit your revision, log on to https://www.editorialmanager.com/pone/ and select the 'Submissions Needing Revision' folder to locate your manuscript file.

- A letter that responds to each point raised by the academic editor and reviewer(s). You should upload this letter as a separate file labeled 'Response to Reviewers'.
- A marked-up copy of your manuscript that highlights changes made to the original version. You should upload this as a separate file labeled 'Revised Manuscript with Track Changes'.
- An unmarked version of your revised paper without tracked changes. You should upload this as a separate file labeled 'Manuscript'.

If you would like to make changes to your financial disclosure, please include your updated statement in your cover letter. Guidelines for resubmitting your figure files are available below the reviewer comments at the end of this letter.

If applicable, we recommend that you deposit your laboratory protocols in protocols.io to enhance the reproducibility of your results. Protocols.io assigns your protocol its own identifier (DOI) so that it can be cited independently in the future. For instructions see: https://journals.plos.org/plosone/s/submission-guidelines#loc-laboratory-protocols. Additionally, PLOS ONE offers an option for publishing peer-reviewed Lab Protocol articles, which describe protocols hosted on protocols.io. Read more information on sharing protocols at https://plos.org/protocols?utm_medium=editorial-email&utm_source=authorletters&utm_campaign=protocols.

As the corresponding author, your ORCID iD is verified in the submission system and will appear in the published article. PLOS supports the use of ORCID, and we encourage all coauthors to register for an ORCID iD and use it as well. Please encourage your coauthors to verify their ORCID iD within the submission system before final acceptance, as unverified ORCID iDs will not appear in the published article. *Only*  the individual author can complete the verification step; PLOS staff *cannot*  verify ORCID iDs on behalf of authors.

We look forward to receiving your revised manuscript.

Kind regards,

Shengwei Sun, Ph.D.

Academic Editor

PLOS One

Journal Requirements:

When submitting your revision, we need you to address these additional requirements.

1. Please ensure that your manuscript meets PLOS ONE's style requirements, including those for file naming. The PLOS ONE style templates can be found at

https://journals.plos.org/plosone/s/file?id=wjVg/PLOSOne_formatting_sample_main_body.pdf and

https://journals.plos.org/plosone/s/file?id=ba62/PLOSOne_formatting_sample_title_authors_affiliations.pdf

2. Thank you for stating the following in the Acknowledgments Section of your manuscript:

“H.J. and C.T.S. acknowledge funding by the German Research Foundation (Deutsche Forschungsgemeinschaft e.V. (DFG)) in the frame of CRC1423 “Structural Dynamics of GPCR Activation and Signaling” (project number 421152132 and project A02). Open access publication was funded by the Open Access Publishing Fund of Leipzig University supported by the German Research Foundation within the program Open Access Publication Funding.”

We note that you have provided funding information that is not currently declared in your Funding Statement. However, funding information should not appear in the Acknowledgments section or other areas of your manuscript. We will only publish funding information present in the Funding Statement section of the online submission form.

Please remove any funding-related text from the manuscript and let us know how you would like to update your Funding Statement. Currently, your Funding Statement reads as follows:

“H.J. and C.T.S. acknowledge funding by the German Research Foundation (Deutsche Forschungsgemeinschaft e.V. (DFG)) in the frame of CRC1423 “Structural Dynamics of GPCR Activation and Signaling” (project number 421152132 and project A02). Open access publication was funded by the Open Access Publishing Fund of Leipzig University supported by the German Research Foundation within the program Open Access Publication Funding.”

Please include your amended statements within your cover letter; we will change the online submission form on your behalf.

3. Thank you for stating the following financial disclosure:

“H.J. and C.T.S. acknowledge funding by the German Research Foundation (Deutsche Forschungsgemeinschaft e.V. (DFG)) in the frame of CRC1423 “Structural Dynamics of GPCR Activation and Signaling” (project number 421152132 and project A02). Open access publication was funded by the Open Access Publishing Fund of Leipzig University supported by the German Research Foundation within the program Open Access Publication Funding.”

Please state what role the funders took in the study.  If the funders had no role, please state: "The funders had no role in study design, data collection and analysis, decision to publish, or preparation of the manuscript."

If this statement is not correct you must amend it as needed.

Please include this amended Role of Funder statement in your cover letter; we will change the online submission form on your behalf.

4. In the online submission form, you indicated that your data will be submitted to a repository upon acceptance.  We strongly recommend all authors deposit their data before acceptance, as the process can be lengthy and hold up publication timelines. Please note that, though access restrictions are acceptable now, your entire minimal  dataset will need to be made freely accessible if your manuscript is accepted for publication. This policy applies to all data except where public deposition would breach compliance with the protocol approved by your research ethics board. If you are unable to adhere to our open data policy, please kindly revise your statement to explain your reasoning and we will seek the editor's input on an exemption.

5. We note that you have included the phrase “data not shown” in your manuscript. Unfortunately, this does not meet our data sharing requirements. PLOS does not permit references to inaccessible data. We require that authors provide all relevant data within the paper, Supporting Information files, or in an acceptable, public repository. Please add a citation to support this phrase or upload the data that corresponds with these findings to a stable repository (such as Figshare or Dryad) and provide and URLs, DOIs, or accession numbers that may be used to access these data. Or, if the data are not a core part of the research being presented in your study, we ask that you remove the phrase that refers to these data.

6. We notice that your supplementary figures and tables are included in the manuscript file. Please remove them and upload them with the file type 'Supporting Information'. Please ensure that each Supporting Information file has a legend listed in the manuscript after the references list.

7. If the reviewer comments include a recommendation to cite specific previously published works, please review and evaluate these publications to determine whether they are relevant and should be cited. There is no requirement to cite these works unless the editor has indicated otherwise.

[Note: HTML markup is below. Please do not edit.]

Reviewers' comments:

Reviewer's Responses to Questions

**Comments to the Author**

1. Is the manuscript technically sound, and do the data support the conclusions?

Reviewer #1: Partly

Reviewer #2: Yes

**********

2. Has the statistical analysis been performed appropriately and rigorously?

Reviewer #1: No

Reviewer #2: Yes

**********

3. Have the authors made all data underlying the findings in their manuscript fully available?

The PLOS Data policy

Reviewer #1: Yes

Reviewer #2: No

**********

4. Is the manuscript presented in an intelligible fashion and written in standard English?

Reviewer #1: Yes

Reviewer #2: Yes

**********

Reviewer #1: General Assessment

This manuscript presents a timely and well-scoped benchmark of deep learning methods for GPCR-targeting peptide prediction and design, framed around the distinction between sampling and scoring. The experimental scope is commendable, including 124 complexes and large-scale generation campaigns. The central finding — that current confidence metrics are insufficiently calibrated for peptide-placement accuracy and may assign misleadingly high confidence to incorrect poses — is important and relevant to practical design workflows. However, several issues should be addressed before the conclusions can be considered robust. The most critical concern is insufficient separation of memorized or training-exposed examples from genuinely prospective predictions, which makes headline performance numbers difficult to interpret. Additional methodological clarification and supplementary stratified analyses would substantially strengthen the manuscript.

Recommendation: Major Revision

Major Concerns

1. Training data leakage must be addressed in the main text

The authors acknowledge that endothelin/sarafotoxin complexes were present in the training sets and that performance degrades for post-cutoff structures (Supplementary Fig. S1, p < 0.001). However, the main results are not stratified by training-set membership or structural redundancy. The bimodal DockQ distribution is consistent with possible memorization and should be analyzed directly. Headline performance numbers, such as the reported Boltz-2 median DockQ and BoltzGen generation precision, should therefore be qualified until in-training and post-cutoff examples are separated. The authors should: (a) annotate main figures by training-set membership, (b) report separate summary statistics for in-training, homologous, and post-cutoff structures, and (c) qualify abstract-level performance claims accordingly. Both aggregate and stratified numbers are useful, but the prospective performance estimate should be clearly distinguished from potentially training-exposed performance. If the high-DockQ mode is enriched for in-training or closely homologous complexes, this should be explicitly stated.

2. PAE aggregation method must be specified

The paper identifies confidence miscalibration or overconfidence in incorrect peptide placements as a central problem, but does not specify whether PAE values reported are min, mean, or median over the inter-chain block. This is a critical methodological detail: min-PAE will be optimistically biased for peptides where even one residue is well-placed, while mean-PAE is sensitive to outlier misplacements. The aggregation method should be clearly stated in Methods, and ideally a supplementary comparison of aggregation strategies should be provided.

3. Nanobodies should be separated from peptide statistics

The dataset includes eight nanobody/sdAb complexes alongside short peptides and peptide hormones. Nanobodies have a stable β-sandwich scaffold that fundamentally changes the prediction task. This is not merely a size issue; it changes the physical problem from flexible peptide docking/design to prediction of a structured protein-domain interface. DockQ, fnat, and iRMSD behave differently for structured proteins vs. flexible peptides, and mixing them may distort aggregate statistics unless analyzed separately. Results should be stratified by size class (e.g., peptides ≤ 50 AA vs. protein ligands > 50 AA vs. nanobodies), and the abstract should state the dataset composition explicitly.

4. The design workflow needs a diagram and parameter documentation

For a benchmark paper, reproducibility is paramount. Critical parameters are currently scattered across Methods and Supplementary materials. The paper should include: (a) a main-text pipeline diagram showing the end-to-end workflow for each method, and (b) a consolidated table of all method-specific parameters with values used. Hotspot residue identifiers should be listed explicitly (not only shown in Supplementary Fig. S4). ProteinMPNN was apparently used at temperature 0.05, which is near-deterministic. The authors should justify this choice and discuss whether broader sequence sampling would alter the rescue analysis.

5. ProteinMPNN “rescue” requires a circularity caveat

The observation that ProteinMPNN sequence redesign improves DockQ scores is measured by re-predicting with the same structure prediction tools. This creates a circularity risk: MPNN produces sequences that structure predictors find easier to fold, not necessarily sequences that would physically bind. The improvement should be described as improved in silico recoverability or predicted placement, not as evidence of physical rescue or binding. The authors should explicitly state that independent physics-based validation would be needed to confirm binding.

6. GPCR terminology correction

Line 93: GPCRs are described as “the largest group of proteins in the human genome.” This is inaccurate or at least imprecise — GPCRs are generally described as the largest family of membrane receptors, not the largest group of proteins overall. Zinc-finger proteins, for example, are often considered a larger protein superfamily. Please correct.

Additional Recommendations

7. Top-k success metrics

The paper would benefit greatly from practical design-workflow metrics: best-of-N DockQ, top-1 and top-5 by confidence, and enrichment of acceptable designs among top-ranked candidates. The current aggregate distributions obscure the question practitioners care about: how many predictions are needed to find one good candidate, and can confidence identify it?

8. Receptor conformation awareness

While the authors appropriately use holo templates from the same PDB entry, it would be helpful to note in the Discussion that real design campaigns will not have access to the cognate holo receptor structure. A brief discussion of how receptor conformational uncertainty (active vs. inactive state, G-protein vs. arrestin stabilization, engineered constructs) affects the transferability of these benchmark results would strengthen the paper’s practical relevance.

9. Membrane context for generation targets

The paper notes that many RFdiffusion3 designs are placed outside the binding pocket in contact with membrane-spanning residues (84.5% for AT2). However, no formal membrane boundary is defined. For the three generation targets, adding OPM-based membrane planes and classifying failures as “membrane-incompatible” vs. “wrong pocket” would strengthen the analysis.

10. Peptide chemistry stratification

The dataset mixes linear, disulfide-constrained, and cyclic peptides. Constrained peptides have reduced conformational entropy and may be systematically easier to predict. A supplementary stratification by constraint class would be informative.

11. DockQ supplementation

DockQ does not capture pharmacophore-level correctness. For the three generation targets, supplementary analysis of peptide N/C-terminal orientation and key hotspot contact recovery would connect the structural metrics to biological function.

12. Steric clashes and refinement

Clash rates are computed on raw outputs without minimization (noted in Fig. 6 caption). Given that the authors recommend Rosetta refinement in the Discussion, relaxing a small representative subset (e.g., 100 structures per method) and reporting the change in clash rate would substantially strengthen the practical relevance of the benchmark.

Reviewer #2: Major revisions

1. I think tha authors should include models and design in the zenodo, not only the PDBs in the dataset.

Minor revisions

1. Please specify the versions of all software packages and tools used in the study. In addition, clarify the programming language(s) used for custom scripts.

2. The manuscript mentions 124 complexes; however, it appears that some receptors are represented by multiple PDB entries. Please clarify how redundant or highly similar receptor structures were handled in the analysis. For example, structures such as 7SK3 and 7SK4 are very similar. Were both included independently? If so, how was potential bias or overlap in the comparison of generated designs addressed when the receptor is repeated?

3. It would be helpful to include an additional column in Supplementary Table S1 indicating the GPCR class of each receptor for easier interpretation of the dataset.

4. In figure 1, the manuscript states that were used 76 Class A ligands plus 31 Class B1 ligands, which sum to 107 rather than 124. Please clarify this discrepancy, as I may have misunderstood the dataset composition.

5. In Figure 2, I suggest including an additional plot for LRMSD to complement the current structural evaluation metrics.

6. For the DockQ analysis shown in Figure 3, it would be useful to include representative structural examples illustrating extreme cases. For instance, showing one example with very low DockQ and another with broader interface agreement would help readers better understand how iRMSD and FNAT contribute to the final DockQ values.

7. I believe the conclusions section would benefit from a more explicit discussion regarding scoring and evaluation metrics. At present, the take-home message about which scoring approaches are most informative or reliable remains somewhat unclear.

8. In the “Comparing the Sampling of BindCraft, BoltzGen and RFdiffusion3” section, it would also be interesting to evaluate sequence or structural recovery metrics and discuss these results in the conclusions. Are the designs similar to the native peptides? This could provide additional insight into how well the generated peptides preserve relevant interaction features or binding determinants.

**********

what does this mean?). If published, this will include your full peer review and any attached files.

If you choose “no”, your identity will remain anonymous but your review may still be made public.

**Do you want your identity to be public for this peer review?**  For information about this choice, including consent withdrawal, please see our Privacy Policy

Reviewer #1: No

Reviewer #2: **Yes:**  Gabriel Ernesto Jara

**********

[NOTE: If reviewer comments were submitted as an attachment file, they will be attached to this email and accessible via the submission site. Please log into your account, locate the manuscript record, and check for the action link "View Attachments". If this link does not appear, there are no attachment files.]

To ensure your figures meet our technical requirements, please review our figure guidelines: https://journals.plos.org/plosone/s/figures

You may also use PLOS’s free figure tool, NAAS, to help you prepare publication quality figures: https://journals.plos.org/plosone/s/figures#loc-tools-for-figure-preparation.

NAAS will assess whether your figures meet our technical requirements by comparing each figure against our figure specifications.

# Author comment: Author response to Decision Letter 1

**DOI:** 10.1371/journal.pone.0355549.r003

1 Jul 2026

Please note that all line locations refer to the markup version of the manuscript.

Response to Reviewer 1:

1. Training data leakage must be addressed in the main text

“The authors acknowledge that endothelin/sarafotoxin complexes were present in the training sets and that performance degrades for post-cutoff structures (Supplementary Fig. S1, p < 0.001). However, the main results are not stratified by training-set membership or structural redundancy. The bimodal DockQ distribution is consistent with possible memorization and should be analyzed directly. Headline performance numbers, such as the reported Boltz-2 median DockQ and BoltzGen generation precision, should therefore be qualified until in-training and post-cutoff examples are separated. The authors should: (a) annotate main figures by training-set membership, (b) report separate summary statistics for in-training, homologous, and post-cutoff structures, and (c) qualify abstract-level performance claims accordingly. Both aggregate and stratified numbers are useful, but the prospective performance estimate should be clearly distinguished from potentially training-exposed performance. If the high-DockQ mode is enriched for in-training or closely homologous complexes, this should be explicitly stated.”

Respose:

We want to thank the reviewer for this highly relevant comment. We agree that we did not elaborate sufficiently on the effect of training set inclusion and that our statements raised more questions than they did answer. In order to address this, we stratified our dataset by release date of the specific PDB IDs as well as homologous deposits of the identical receptor-peptide pair. We decided to keep the initial Fig. 2 (now Fig. 3) for visual clarity and summarized the DockQ scores for pre and post cutoff PDB IDs which were added to the Supplement as S3 Fig. We further edited the manuscript accordingly to address this.

Edit locations:

Page 12, lines 239-256;

Page 30, lines 534-538 in the revised, marked-up manuscript.

S3 Fig.

2. PAE aggregation method must be specified

“The paper identifies confidence miscalibration or overconfidence in incorrect peptide placements as a central problem, but does not specify whether PAE values reported are min, mean, or median over the inter-chain block. This is a critical methodological detail: min-PAE will be optimistically biased for peptides where even one residue is well-placed, while mean-PAE is sensitive to outlier misplacements. The aggregation method should be clearly stated in Methods, and ideally a supplementary comparison of aggregation strategies should be provided.”

Respose:

Thank you for highlighting this methodological detail. We edited our manuscript to clarify that we averaged PAE values by taking the mean over all PAE values for the respective chain subset.

Location:

Page 19, lines 339-340 in the revised, marked-up manuscript.

3. Nanobodies should be separated from peptide statistics

“The dataset includes eight nanobody/sdAb complexes alongside short peptides and peptide hormones. Nanobodies have a stable β-sandwich scaffold that fundamentally changes the prediction task. This is not merely a size issue; it changes the physical problem from flexible peptide docking/design to prediction of a structured protein-domain interface. DockQ, fnat, and iRMSD behave differently for structured proteins vs. flexible peptides, and mixing them may distort aggregate statistics unless analyzed separately. Results should be stratified by size class (e.g., peptides ≤ 50 AA vs. protein ligands > 50 AA vs. nanobodies), and the abstract should state the dataset composition explicitly.”

Respose:

We want to thank the reviewer for this objection. While we remain confident with our initial assumption that peptides, hormones and the loops of nAbs share comparable interaction characteristics with GPCRs, we revisited the data and confirm that the prediction tools indeed perform differently for larger ligands. We therefore modified Figure 5 and added Supplementary Fig. S2. We adapted the manuscript accordingly.

Location:

Page 7, lines 158-160;

Page 11, lines 223-227;

Page 13, lines 271-275 in the revised, marked-up manuscript.

4. The design workflow needs a diagram and parameter documentation

“For a benchmark paper, reproducibility is paramount. Critical parameters are currently scattered across Methods and Supplementary materials. The paper should include: (a) a main-text pipeline diagram showing the end-to-end workflow for each method, and (b) a consolidated table of all method-specific parameters with values used. Hotspot residue identifiers should be listed explicitly (not only shown in Supplementary Fig. S4). ProteinMPNN was apparently used at temperature 0.05, which is near-deterministic. The authors should justify this choice and discuss whether broader sequence sampling would alter the rescue analysis.”

Respose:

We appreciate the comments regarding methodological clarity.

(a) We agree that a workflow diagram significantly improves clarity of the manuscript. We added a pipeline diagram to visualize the workflow of the two parts of our benchmark as Figure 1.

Location:

Page 6, lines 137-148 in the revised, marked-up manuscript.

(b) We agree with the reviewer that for the sake of reproducibility, method parameters need to be clearly stated. Instead of a table, which became too large to be presented visually appealing, we opted to revise the Methods section as we purposefully kept the majority of settings default. We further highlighted those parameters that we modified and refer to the methods documentation as well as our exemplary scripts in the accompanying zenodo.

For these changes see:

Page 32, lines 599-602 and the remainder of the Methods section in the revised, marked-up manuscript.

Additionally, we agree that the previous hotspot-related figure was insufficient. We added S2 Table containing the hotspot residue identifiers and their respective Ballesteros-Weinstein numbering in the SI as S2 Table

Further, the reviewer raises a very valid point as sequence composition is the final determining factor in designing novel peptides. The reasoning behind a temperature of 0.05 was that increasing diversity would require a larger number of sequences to be evaluated, which would shift the focus of the benchmark. We expanded our discussion with respect to ProteinMPNN in our manuscript.

For these changes please refer to Page 26, line 463 and Page 30, lines 552-558

5. ProteinMPNN “rescue” requires a circularity caveat

“The observation that ProteinMPNN sequence redesign improves DockQ scores is measured by re-predicting with the same structure prediction tools. This creates a circularity risk: MPNN produces sequences that structure predictors find easier to fold, not necessarily sequences that would physically bind. The improvement should be described as improved in silico recoverability or predicted placement, not as evidence of physical rescue or binding. The authors should explicitly state that independent physics-based validation would be needed to confirm binding.”

Respose:

Thank you for pointing out this critical detail inherent in virtually all currently available ML-based pipelines. We agree that our initial wording needed improvement and we adapted the discussion respectively to include these arguments (see Page 29, lines 552-558)

6. GPCR terminology correction

“Line 93: GPCRs are described as “the largest group of proteins in the human genome.” This is inaccurate or at least imprecise — GPCRs are generally described as the largest family of membrane receptors, not the largest group of proteins overall. Zinc-finger proteins, for example, are often considered a larger protein superfamily. Please correct.”

Respose:

We thank the reviewer for highlighting this inaccuracy. This was indeed not the intended phrasing and we adapted the manuscript accordingly.

Location:

Page 4, line 97

7. Top-k success metrics

“The paper would benefit greatly from practical design-workflow metrics: best-of-N DockQ, top-1 and top-5 by confidence, and enrichment of acceptable designs among top-ranked candidates. The current aggregate distributions obscure the question practitioners care about: how many predictions are needed to find one good candidate, and can confidence identify it?”

Respose:

We appreciate this suggestion and confirm that reporting metrics of top-1 candidates holds great value and that allowed us to strengthen our benchmark. We adapted the corresponding Figures (now Fig. 6 and S5 Fig) and addressed it in the manuscript. We further discuss that a general number of predictions required to find a good candidate cannot be sufficiently provided within the scope of this paper as these numbers are highly target- and method-dependent and we would argue that 50 predictions already is a sufficiently large number.

Location:

Page 18, lines 325-333

Page 28, lines 497-499

Page 30, line 539

8. Receptor conformation awareness

“While the authors appropriately use holo templates from the same PDB entry, it would be helpful to note in the Discussion that real design campaigns will not have access to the cognate holo receptor structure. A brief discussion of how receptor conformational uncertainty (active vs. inactive state, G-protein vs. arrestin stabilization, engineered constructs) affects the transferability of these benchmark results would strengthen the paper’s practical relevance.”

Respose:

We want to thank the reviewer for pointing out this crucial fact regarding GPCR drug discovery. We connect the conformational uncertainty with the likelihood of memorization and discuss how these two aspects might prevent succesfull results in real drug design campaigns.

Location:

Page 30, lines 534-538

9. Membrane context for generation targets

“The paper notes that many RFdiffusion3 designs are placed outside the binding pocket in contact with membrane-spanning residues (84.5% for AT2). However, no formal membrane boundary is defined. For the three generation targets, adding OPM-based membrane planes and classifying failures as “membrane-incompatible” vs. “wrong pocket” would strengthen the analysis.”

Respose:

We want to thank the reviewer for this relevant remark. We revised our initial method to determine the placement of designed peptides, i.e. through the distance to hotspots and RMSD to native and concluded that these were insufficient in precisely determining the location, i.e. “in pocket” or “in membrane”. We therefore performed a more thorough contact analysis and were able to update our previous percentages. We adapted the manuscript accordingly. The updated numbers and a depiction of the OPM membrane definitions are summarized in Supplementary Fig. S5.

Location:

Page 24, lines 419-421

10. Peptide chemistry stratification

“The dataset mixes linear, disulfide-constrained, and cyclic peptides. Constrained peptides have reduced conformational entropy and may be systematically easier to predict. A supplementary stratification by constraint class would be informative.”

Respose:

Thank you for this comment. We stratified by cyclic constraint and added S4 Fig and addressed it in the manuscript.

Location:

Page 11, lines 227-229

Added S4 Fig to the supplement

11. DockQ supplementation

“DockQ does not capture pharmacophore-level correctness. For the three generation targets, supplementary analysis of peptide N/C-terminal orientation and key hotspot contact recovery would connect the structural metrics to biological function.”

Respose:

We agree with the reviewer that the pharmacophore-level correctness is a highly relevant piece of information, especially with regard to actual drug discovery campaigns. However, during our initial analysis we realized that N/C-terminal orientation (such as flipped termini) is difficult to generalize sufficiently, as designs often did not end up with a linear N- or C-terminal entry deep into the pocket, especially for the cyclic representative Sarafotoxin S6b. We further believe that in true de novo peptide design campaigns novel peptide orientations are desired and their correctness cannot be solely described through terminal orientation.

12. Steric clashes and refinement

“Clash rates are computed on raw outputs without minimization (noted in Fig. 6 caption). Given that the authors recommend Rosetta refinement in the Discussion, relaxing a small representative subset (e.g., 100 structures per method) and reporting the change in clash rate would substantially strengthen the practical relevance of the benchmark.”

Respose:

We fully agree with the reviewer that the outcome of Rosetta refinement is of great interest. We opted to leave those calculations out of this benchmark for different reasons: a) we wanted to keep the benchmark in the scope of ML-based methods. b) the strength of Rosetta refinement has been reported extensively before and adding it to this benchmark would not provide significant new insights. c) while we expect that Rosetta resolves both minor and severe clashes, the actual extent of this is highly dependent on the protocols used, i.e. minimization vs. relaxation, restricting vs. allowing movement of the target or the use of membrane/peptide specific scoring functions. In order to still provide Rosetta specific instructions we adapted the manuscript and refer to a selection of Rosetta-specific literature.

Location:

Page 31, lines 571-572.

Response to Reviewer 2:

1. “I think tha authors should include models and design in the zenodo, not only the PDBs in the dataset.”

Respose:

We agree with the reviewer that in order to guarantee full transparency, output data has to be included in the zenodo. We adapted the repositories respectively and also appended it with exemplary scripts necessary for reproduction.

1. “Please specify the versions of all software packages and tools used in the study. In addition, clarify the programming language(s) used for custom scripts.”

Respose:

We want to thank the reviewer for pointing out the lack of documentation regarding the software package version used in this work. We amended the method section to clarify this. Since the main methods used in this work depend on a large number of supplementary packages, we referred to the respective installation protocols.

Location:

Page 37, lines 701-721

2. “The manuscript mentions 124 complexes; however, it appears that some receptors are represented by multiple PDB entries. Please clarify how redundant or highly similar receptor structures were handled in the analysis. For example, structures such as 7SK3 and 7SK4 are very similar. Were both included independently? If so, how was potential bias or overlap in the comparison of generated designs addressed when the receptor is repeated?”

Respose:

We want to thank the reviewer for this correct observation. Based on this comment, we revisited our dataset composition and identified further occurrences of duplicate entries that went unnoticed previously due to inconsistent naming in the GPCRdb. Keeping these would scew statistics and we therefore removed duplicates and updated the downstream analyses and figures. This reduced the dataset from 124 dimers to 113 dimers. Accordingly, Figure 2 was adapted to include these changes.

Location:

Page 1, lines 33-34

Page 9, line 185 (Fig.2)

3. “It would be helpful to include an additional column in Supplementary Table S1 indicating the GPCR class of each receptor for easier interpretation of the dataset.”

Respose:

We agree that this is helpful information and added the column to Supplementary Table S1.

Location:

Supporting Information, S1 Table.

4. “In figure 1, the manuscript states that were used 76 Class A ligands plus 31 Class B1 ligands, which sum to 107 rather than 124. Please clarify this discrepancy, as I may have misunderstood the dataset composition.”

Respose:

Thank you for addressing this issue. We revised the manuscript to emphasize the many-to-many relationship of GPCRs and their peptides, where one peptide can bind to multiple GPCRs and one GPCR can accept binding of mu

Attachment Submitted filename: Response_to_Reviewers.pdf

# Peer review: Decision Letter 1

**DOI:** 10.1371/journal.pone.0355549.r004

**Authors:** Shengwei Sun

15 Jul 2026

*De Novo*

Dear Dr. Junker,

Thank you for submitting your manuscript to PLOS ONE. After careful consideration, we feel that it has merit but does not fully meet PLOS ONE’s publication criteria as it currently stands. Therefore, we invite you to submit a revised version of the manuscript that addresses the points raised during the review process.

Please submit your revised manuscript by Aug 29 2026 11:59PM. If you will need more time than this to complete your revisions, please reply to this message or contact the journal office at plosone@plos.org. When you're ready to submit your revision, log on to https://www.editorialmanager.com/pone/ and select the 'Submissions Needing Revision' folder to locate your manuscript file.

- A letter that responds to each point raised by the academic editor and reviewer(s). You should upload this letter as a separate file labeled 'Response to Reviewers'.
- A marked-up copy of your manuscript that highlights changes made to the original version. You should upload this as a separate file labeled 'Revised Manuscript with Track Changes'.
- An unmarked version of your revised paper without tracked changes. You should upload this as a separate file labeled 'Manuscript'.

If you would like to make changes to your financial disclosure, please include your updated statement in your cover letter. Guidelines for resubmitting your figure files are available below the reviewer comments at the end of this letter.

If applicable, we recommend that you deposit your laboratory protocols in protocols.io to enhance the reproducibility of your results. Protocols.io assigns your protocol its own identifier (DOI) so that it can be cited independently in the future. For instructions see: https://journals.plos.org/plosone/s/submission-guidelines#loc-laboratory-protocols. Additionally, PLOS ONE offers an option for publishing peer-reviewed Lab Protocol articles, which describe protocols hosted on protocols.io. Read more information on sharing protocols at https://plos.org/protocols?utm_medium=editorial-email&utm_source=authorletters&utm_campaign=protocols.

As the corresponding author, your ORCID iD is verified in the submission system and will appear in the published article. PLOS supports the use of ORCID, and we encourage all coauthors to register for an ORCID iD and use it as well. Please encourage your coauthors to verify their ORCID iD within the submission system before final acceptance, as unverified ORCID iDs will not appear in the published article. *Only*  the individual author can complete the verification step; PLOS staff *cannot*  verify ORCID iDs on behalf of authors.

We look forward to receiving your revised manuscript.

Kind regards,

Shengwei Sun, Ph.D.

Academic Editor

PLOS One

Journal Requirements:

If the reviewer comments include a recommendation to cite specific previously published works, please review and evaluate these publications to determine whether they are relevant and should be cited. There is no requirement to cite these works unless the editor has indicated otherwise.

Please review your reference list to ensure that it is complete and correct. If you have cited papers that have been retracted, please include the rationale for doing so in the manuscript text, or remove these references and replace them with relevant current references. Any changes to the reference list should be mentioned in the rebuttal letter that accompanies your revised manuscript. If you need to cite a retracted article, indicate the article’s retracted status in the References list and also include a citation and full reference for the retraction notice.

[Note: HTML markup is below. Please do not edit.]

Reviewers' comments:

Reviewer's Responses to Questions

**Comments to the Author**

Reviewer #1: All comments have been addressed

Reviewer #2: All comments have been addressed

**********

2. Is the manuscript technically sound, and do the data support the conclusions?

Reviewer #1: Yes

Reviewer #2: Yes

**********

3. Has the statistical analysis been performed appropriately and rigorously?

Reviewer #1: Yes

Reviewer #2: Yes

**********

4. Have the authors made all data underlying the findings in their manuscript fully available?

The PLOS Data policy

Reviewer #1: Yes

Reviewer #2: Yes

**********

5. Is the manuscript presented in an intelligible fashion and written in standard English?

Reviewer #1: Yes

Reviewer #2: Yes

**********

Reviewer #1: Dear Authors,

Thanks for the careful revision. The new workflow figure, the training-cutoff and ligand-size stratifications, the hotspot table, the updated membrane analysis, and the clearer discussion of memorization have all made the paper noticeably stronger. It is clearly a better paper now.

Most of my original concerns are now well addressed. I have only one remaining practical issue.

In the Discussion you recommend Rosetta refinement to resolve clashes. While I accept that you kept new calculations out of scope, the XML protocol file (or a fully reproducible RosettaScripts protocol) does not appear to be available in the Zenodo repository or supplementary materials. For a benchmark paper that already emphasizes reproducibility, it would be very helpful if you could either deposit the exact XML script you have in mind or provide a clear, ready-to-run protocol (ideally with the ref2015 energy function and appropriate membrane terms).

It is also necessary to make sure the right citations are used so in the methods for rosetta the ref2015 should be properly cited. Citing only the general development papers is not enough for readers to actually reproduce or adapt the suggested step.

Please check that ALL scripts and files are provided for fellow researchers.

With that those addition I’m happy to recommend acceptance.

Best regards,

Reviewer 1

Reviewer #2: The total complex are 113. However, the sum of classes is not equal to 113 (58 class A +14 class B1). The author should explain this or make a correction in the text

**********

what does this mean?). If published, this will include your full peer review and any attached files.

If you choose “no”, your identity will remain anonymous but your review may still be made public.

**Do you want your identity to be public for this peer review?**  For information about this choice, including consent withdrawal, please see our Privacy Policy

Reviewer #1: No

Reviewer #2: No

**********

[NOTE: If reviewer comments were submitted as an attachment file, they will be attached to this email and accessible via the submission site. Please log into your account, locate the manuscript record, and check for the action link "View Attachments". If this link does not appear, there are no attachment files.]

To ensure your figures meet our technical requirements, please review our figure guidelines: https://journals.plos.org/plosone/s/figures

You may also use PLOS’s free figure tool, NAAS, to help you prepare publication quality figures: https://journals.plos.org/plosone/s/figures#loc-tools-for-figure-preparation.

NAAS will assess whether your figures meet our technical requirements by comparing each figure against our figure specifications.

# Author comment: Author response to Decision Letter 2

**DOI:** 10.1371/journal.pone.0355549.r005

20 Jul 2026

Please note that all line locations refer to the markup version of the manuscript.

Response to Reviewer 1:

“In the Discussion you recommend Rosetta refinement to resolve clashes. While I accept

that you kept new calculations out of scope, the XML protocol file (or a fully reproducible

RosettaScripts protocol) does not appear to be available in the Zenodo repository or

supplementary materials. For a benchmark paper that already emphasizes reproducibility, it

would be very helpful if you could either deposit the exact XML script you have in mind or

provide a clear, ready-to-run protocol (ideally with the ref2015 energy function and

appropriate membrane terms).

It is also necessary to make sure the right citations are used so in the methods for rosetta

the ref2015 should be properly cited. Citing only the general development papers is not

enough for readers to actually reproduce or adapt the suggested step.

Please check that ALL scripts and files are provided for fellow researchers.

With that those addition I’m happy to recommend acceptance.”

Response:

We note the reviewer's wish for a reproducible RosettaScripts XML protocol and the

corresponding citation for the ref2015 energy function. An exemplary XML script, configured

with the ref2015 energy function and applicable membrane terms, has been added to our

zenodo. Furthermore, the manuscript has been revised to include the specific citation for the

ref2015 energy function.

Response to Reviewer 2:

“The total complex are 113. However, the sum of classes is not equal to 113 (58 class A +14

class B1). The author should explain this or make a correction in the text”

Response:

Dear Reviewer 2, we would kindly refer to page 7, lines 164-174 in the manuscript where we

highlighted the many-to-many relationship between receptors and their cognate peptides:

"[...] The final dataset consists of 74 peptides binding to 58 class A receptors and 26 peptides

binding to 14 class B1 receptors, reflecting the promiscuous many-to-many binding

relationship that is characteristic for many GPCRs and their endogenous peptides (Fig. 2A).

Prominent examples for this promiscuity in our dataset are the Glucagon-like peptide-1

receptor (GLP1R), amounting to five unique GLP1R-ligand complexes, or the Endothelin

receptor type B (ETB) with four unique ETB-ligand complexes. Promiscuous ligands on the

other side usually occur in the dataset fewer in numbers and mostly within a subfamily of

receptors, e.g. Ang II binding to both Angiotensin II receptor type 1 (AT1) and type 2 (AT2)

or Substance P binding to both Tachykinin receptor 1 (NK1) and Mas-related G protein

coupled receptor member X2 (MRGPRX2). [...]"

Attachment Submitted filename: Response_to_Reviewers_v3.pdf

# Editor report: Decision Letter 2

**DOI:** 10.1371/journal.pone.0355549.r006

**Authors:** Shengwei Sun

23 Jul 2026

Assessment of Generative *De Novo*  Peptide Design Methods for G Protein-Coupled Receptors

PONE-D-26-13282R2

Dear Dr. Junker,

We’re pleased to inform you that your manuscript has been judged scientifically suitable for publication and will be formally accepted for publication once it meets all outstanding technical requirements.

Within one week, you’ll receive an e-mail detailing the required amendments. When these have been addressed, you’ll receive a formal acceptance letter and your manuscript will be scheduled for publication.

An invoice will be generated when your article is formally accepted. Please note, if your institution has a publishing partnership with PLOS and your article meets the relevant criteria, all or part of your publication costs will be covered. Please make sure your user information is up-to-date by logging into Editorial Manager at Editorial Manager® and clicking the ‘Update My Information' link at the top of the page. For questions related to billing, please contact billing support.

If your institution or institutions have a press office, please notify them about your upcoming paper to help maximize its impact. If they’ll be preparing press materials, please inform our press team as soon as possible -- no later than 48 hours after receiving the formal acceptance. Your manuscript will remain under strict press embargo until 2 pm Eastern Time on the date of publication. For more information, please contact onepress@plos.org.

Kind regards,

Shengwei Sun, Ph.D.

Academic Editor

PLOS One

Additional Editor Comments (optional):

Reviewers' comments:

# Editor report: Acceptance letter

**DOI:** 10.1371/journal.pone.0355549.r007

**Authors:** Shengwei Sun

PONE-D-26-13282R2

PLOS One

Dear Dr. Schoeder,

I'm pleased to inform you that your manuscript has been deemed suitable for publication in PLOS One. Congratulations! Your manuscript is now being handed over to our production team.

At this stage, our production department will prepare your paper for publication. This includes ensuring the following:

* All references, tables, and figures are properly cited

* All relevant supporting information is included in the manuscript submission,

* There are no issues that prevent the paper from being properly typeset

You will receive further instructions from the production team, including instructions on how to review your proof when it is ready. Please keep in mind that we are working through a large volume of accepted articles, so please give us a few days to review your paper and let you know the next and final steps.

Lastly, if your institution or institutions have a press office, please let them know about your upcoming paper now to help maximize its impact. If they'll be preparing press materials, please inform our press team within the next 48 hours. Your manuscript will remain under strict press embargo until 2 pm Eastern Time on the date of publication. For more information, please contact onepress@plos.org.

You will receive an invoice from PLOS for your publication fee after your manuscript has reached the completed accept phase. If you receive an email requesting payment before acceptance or for any other service, this may be a phishing scheme. Learn how to identify phishing emails and protect your accounts at https://explore.plos.org/phishing.

If we can help with anything else, please email us at customercare@plos.org.

Thank you for submitting your work to PLOS One and supporting open access.

Kind regards,

PLOS ONE Editorial Office Staff

on behalf of

Dr. Shengwei Sun

Academic Editor

PLOS One
